# Supplementary material for: Aggregating forecasts of multiple respiratory pathogens supports more accurate forecasting of influenza-like illness
Source: PLoS Comput Biol. 2020 Oct 22;16(10):e1008301. doi: 10.1371/journal.pcbi.1008301 (PMC7608986; doi:10.1371/journal.pcbi.1008301)
Supplement: S1 Text — (DOCX) [file pcbi.1008301.s001.docx]

**Supplementary material**

**Disaggregation of ILI**

We here derive how ILI can be represented as a linear combination of its contributing viral signals. ILI is a rate that measures the conditional probability a patient seeking medical attention (event $M$) is diagnosed with ILI (event $I$) at time $t$ – $P_{t}(I|M)$. Assume that ILI is attributed to a range of pathogens ${\{V}_{i}\}$ (symptomatic infection by each possible cause of ILI). The ILI rate can be decomposed to

$$P_{t}\left( I | M \right)=\sum_{i} P_{t}(V_{i}|M) (1)$$

where $P_{t}(V_{i}|M)$ is the probability that a patient seeking medical treatment is symptomatically infected with pathogen $V_{i}$ at time $t$. Denote event $T$ as a sample taken from a patient for testing in laboratory. The positivity rate of pathogen $V_{i}$ in laboratory tests is the conditional probability $P_{t}(V_{i}|M,T)$. Bayes’ rule yields

$$P_{t}\left( V_{i} | M,T \right)=P_{t}\left( V_{i} | M \right)\frac{P_{t}\left( T | M,V_{i} \right)}{P_{t}\left( T | M \right)}, (2)$$

in which $P_{t}(T|M)$ is the probability that a patient who visits a doctor for any reason is tested at time $t$, and $P_{t}(T|M,V_{i})$ is the probability that a patient seeking treatment and symptomatically infected by pathogen $V_{i}$ is tested at time $t$. Using this relationship, we have

$$P_{t}\left( I | M \right)=\sum_{i} \frac{P_{t}(T|M)}{P_{t}(T|M,V_{i})}P_{t}(V_{i}|M,T)\equiv\sum_{i} w_{i}\left( t \right)P_{t}\left( V_{i} | M,T \right). (3)$$

Here, the multiplicative factor $w_{i}\left( t \right)=P_{t}\left( T | M \right) /P_{t}\left( T | M,V_{i} \right)$ quantifies the ratio of the probability of undergoing a laboratory test among patients seeking medical attention for any reason to the probability of undergoing a laboratory test among patients seeking medical attention who are infected with pathogen $V_{i}$. Next we show that ILI is a linear combination of the positivity rates of individual ILI-related pathogens. If the multiplicative factor $w_{i}(t)$ is stable over time, we can predict ILI by aggregating forecasts for all contributing pathogens. This establishes the theoretical basis for multi-pathogen ILI forecasting.

To validate the above result, we performed a linear regression of ILI with a constant multiplicative factor $w_{i}$ for each pathogen (S5 Figure). The regression curves agree well with the observed ILI, indicating that the assumption of constant multiplicative factors within a cold and flu season is valid. In contrast, curve fitting using a single-pathogen model leads to substantial discrepancies (S5 Figure, S1 Table).

**Forecasting influenza**

We model influenza transmission using a humidity-forced SIRS model [1]:

$$\frac{dS}{dt}=\frac{N-S-I}{L}-\frac{\beta\left( t \right)IS}{N}, (4)$$

$$\frac{dI}{dt}=\frac{\beta\left( t \right)IS}{N}-\frac{I}{D}. (5)$$

Here $S$, $I$ and $N$ are the susceptible, infected and total population; $L$ is the average duration of immunity; $D$ is the average infectious period; and $\beta\left( t \right)$ is the contact rate at time $t$, modulated by local absolute humidity (AH) conditions through $R_{0}\left( t \right)=\beta\left( t \right)D=\exp\left( a\times q\left( t \right)+b \right)+R_{0min}$, where $R_{0}(t)$ is the basic reproductive number and $q(t)$ is the daily specific humidity, a measure of AH [2,3]. The parameters $a=-180$ and $b=\log(R_{0max}-R_{0min})$ are estimated from laboratory tests of the impact of AH on influenza virus survival ($R_{0max}$ and $R_{0min}$ are the maximal and minimal daily basic reproductive numbers). The absolute humidity (AH) data at national and regional levels were obtained from North American Land Data Assimilation System [4]. A daily AH climatology of conditions averaged over a 24-year period from 1979 to 2002 was used.

In the humidity-driven SIRS model, we use the fraction of newly infected people among the whole population $P_{t}(V_{i})$ to estimate the positivity rate in laboratory testing $P_{t}\left( V_{i} | M,T \right)$. Because the mechanistic model describes influenza transmission in the general population and the positivity rate is defined for a subset of patients who get tested, we need to map the quantity $P_{t}(V_{i})$ in the model to the observation $P_{t}\left( V_{i} | M,T \right)$. Using Bayes’ rule, we have

$$P_{t}\left( V_{i} \right)=\frac{P_{t}\left( T,M \right)}{P_{t}\left( T,M | V_{i} \right)}P_{t}\left( V_{i} | M,T \right)\equiv\gamma_{i}P_{t}\left( V_{i} | M,T \right). (6)$$

A scaling parameter $\gamma_{i}$ is thus needed to perform the transformation. Note that, $\gamma_{i}$ is different from the multiplicative factor $w_{i}$. Here, $P_{t}\left( T,M \right)$ is the probability that an individual in the general population goes to see a doctor and is tested; $P_{t}\left( T,M | V_{i} \right)$ is the conditional probability for those individuals infected by pathogen $V_{i}$.

In implementation, we used model simulations to estimate the scaling parameter $\gamma_{i}$ for each pathogen. Specifically, we ran free simulations using the mechanistic model and computed a distribution of the annual total incidence rate. We then compared the simulated annual incidence rate with the observed annual positivity rate (sum of weekly positivity rates). Here free simulations were generated using initial conditions and model parameters randomly drawn from the prior ranges, representing typical outbreaks generated from the model. Those synthetic outbreaks were used to compute the typical annual incidence rate of model simulations. The scaling parameter $\gamma_{i}$ was estimated as the ratio of the simulated annual incidence rate averaged over 1,000 free simulations with random initialization to the average value of the observed annual positivity rate. With this setting, the mechanistic model is capable of generating outbreaks that in general match observations.

The ensemble forecast for each influenza virus was generated in a hierarchical framework. We first used process-based models to generate an ensemble of forecast trajectories for the unobserved true positivity rate $\bar{v}_{i}(t)$, and then imposed observational errors to obtain the forecast for error-laden observations $y_{i}(t)$ of positivity.

At each forecast week, we inferred the model state using available data via iterated filtering [5]. Here, the Ensemble Adjustment Kalman Filter (EAKF) algorithm [6] with 300 ensemble members was applied iteratively in place of a particle filter [7]. The initial ranges of model variables and parameters were set as follows: $N={10}^{5}$, $S\in\left[ 0.6,0.8 \right]N$, $I\in\left[ 0,0.001 \right]N$, $R_{0max}\in\left[ 1.3, 4 \right]$, $R_{0min}\in\left[ 0.8, 1.3 \right]$, $L\in\left[ 180, 730 \right]$ days, $D\in[2,7]$ days. In the EAKF, the form of observation error variance (OEV) at week $t$ was heuristically set as

$$OEV_{t}=\sigma_{y}^{2}+{v_{i}\left( t \right)}^{2}/k_{y}^{2}, (7)$$

a baseline plus a term related to the positivity rate during the latest week. The parameter $\sigma_{y}$ for each pathogen in each location was set as the standard deviation of the positivity rates that contribute less than 5% to the annual total. The parameter $k_{y}$ was pre-trained using historical data via iterated filtering that includes $k_{y}\in(0,10]$ as an additional parameter in the model state, and kept fixed during forecast. In the iterated filtering algorithm, a total of 10 rounds of EAKF were performed, with the variance of variables/parameters reduced by 10% after each round of the EAKF.

To generate an ensemble of forecast trajectories, we perturbed the fitted model state at the forecast time and projected these perturbed states into the future [8-10]. In particular, we imposed perturbations along the fastest growing direction $e_{1}=(e_{11},e_{12})$ in the *S-I* plane:

$$\left( S,I \right)\to\left( S,I \right)+p\times{(e}_{11}S, e_{12}I), (8)$$

where the perturbation magnitude follows a Gaussian distribution $p\sim\mathcal{N}(0,\sigma_{p}^{2})$. This procedure, named optimal perturbation, aims to generate forecast ensembles with sufficient spread, and has been applied in numerical weather prediction [7,8] and ensemble forecast of influenza [10]. The fastest growing direction is calculated as the eigenvector corresponding to the largest eigenvalue of the linear propagator (see details in Ref. 10).

The perturbation magnitude is determined by the standard deviation $\sigma_{p}$. In prior work we have found that the optimal perturbation magnitude depends on the error growth rate $\lambda_{1}$ (the largest eigenvalue of the linear propagator) [10]: a larger perturbation is needed for dynamics with a slower growth rate $\lambda_{1}$, and a smaller perturbation is sufficient for dynamics with a faster growth rate $\lambda_{1}$. As a result, we assume $\sigma_{p}$ is related to $\lambda_{1}$ in the following form:

$$\sigma_{p}=\sigma_{0}\exp\left( -k_{p}\lambda_{1} \right). (9)$$

Here $k_{p}\geq0$ is a tuning parameter. This form is flexible in that, if $k_{p}=0$, $\sigma_{p}$ is uniform for all forecasts; if $k_{p}>0$, a large error growth rate will lead to a smaller perturbation. Using this perturbation, we generated an ensemble of forecast trajectories: $\left\{ v_{i}^{j}\left( t \right) \right\}$. We model the observed positivity rates as samples from a Gaussian distribution:

$$y_{i}^{j}\left( t \right)\sim\mathcal{N}\left( v_{i}^{j}\left( t \right),\sigma_{y}^{2}+\left( v_{i}^{j}\left( t \right) \right)^{2}/k_{y}^{2} \right), (10)$$

where parameters $\sigma_{y}$ and $k_{y}$ control the observational error variance. Note, if the sampled positivity rate is negative, we set it as zero.

In total, there are four parameters in the hierarchical model: $\sigma_{0}$ and $k_{p}$ for optimal perturbation, and $\sigma_{y}$ and $k_{y}$ for the observational error variance. The parameters $\sigma_{y}$ and $k_{y}$, obtained in advance using iterated filtering, were fixed in the model. The optimal parameters $\sigma_{0}$ and $k_{p}$ were found using a standard simulated annealing (SA) algorithm [11]. The objective was to maximize the average log score for 1- to 4-week ahead prediction. The ranges for these parameters were set as: $\sigma_{0}\in[0.01,1]$ and $k_{p}\in[0,10]$.

For the probabilistic forecasts, the prediction intervals generated by the forecasting system should be well calibrated with the spread of observations (i.e. 50% of observed outcomes fall within the interquartile prediction interval; 95% of observed outcomes fall within the 95% prediction interval; etc.). We evaluated this property using reliability plots. Specifically, we calculated the fraction of observed targets falling within the 25%, 50%, 75% and 100% prediction intervals, and display the relationship between the observed fractions and prediction intervals in S10 Figure. A well-calibrated forecast should have the data points lie on the diagonal line $y=x$. Results in S10 Figure indicate that the near-term forecasts for influenza viruses are well calibrated.

**Forecasting non-influenza pathogens**

Historical records of RSV, PIV12 and PIV3 indicate that these non-influenza pathogens are relatively stable across seasons. As a result, we used a statistical method that leverages their similarity in different seasons. The method of analogues [12] measures the distance from the epidemic curve in the current season to those in other seasons in the same location, and generates forecast as a weighted average of historical trajectories.

For real-time data $v_{i}(1:t_{f})$ and historical records $v_{i,h}(1:t_{f})$ ($h=1,\cdots,l$) up to the forecast time $t_{f}$, the distance is defined as

$$d\left( v_{i}\left( 1:t_{f} \right), v_{i,h}\left( 1:t_{f} \right) \right)=\sum_{t=1}^{t_{f}} \left( v_{i}\left( t \right)-v_{i,h}\left( t \right) \right)^{2}. (11)$$

The forecast curve for $t>t_{f}$ is given by

$$\hat{v}_{i}\left( t \right)=\sum_{h=1}^{l} w_{h}^{d}\left( t_{f} \right)v_{i,h}(t). (12)$$

Here the weight $w_{h}^{d}\left( t_{f} \right)$ is determined by the distance from real-time data $v_{i}\left( 1:t_{f} \right)$ to historical record $v_{i,h}\left( 1:t_{f} \right)$:

$$w_{h}^{d}\left( t_{f} \right)=\frac{d\left( v_{i}\left( 1:t_{f} \right), v_{i,h}\left( 1:t_{f} \right) \right)^{-\alpha}}{\sum_{h=1}^{l} d\left( v_{i}\left( 1:t_{f} \right), v_{i,h}\left( 1:t_{f} \right) \right)^{-\alpha}}. (13)$$

A tunable parameter $\alpha\in[0,1]$ determines how the weight $w_{h}^{d}\left( t_{f} \right)$ depends on distance. This parameter was optimized to reach the minimum forecast error in cross-validation.

We generated forecast trajectories in two steps: 1) sampling $n=1,000$ time series $\left\{ v_{i}^{j} \right\}$ from $l$ historical records according to the weight $w_{h}^{d}\left( t_{f} \right)$; 2) redistributing the trajectories around the mean curve. The $j$th ensemble trajectory is

$$v_{i}^{j}\left( t \right)=\bar{v}_{i}\left( t \right)+c_{v}\left( v_{i}^{j}\left( t \right)-\hat{v}_{i}\left( t \right) \right), (14)$$

where $c_{v}$ controls the spread of the ensemble. We optimized $c_{v}\in[0.5,1.5]$ to achieve the largest log score averaged over 1- to 4-week ahead forecasts. S10 Figure indicates that the optimized forecast ensemble is well calibrated. For the purpose of this retrospective forecast, all time series other than the one being forecast were used as “historical records” (i.e., to forecast the 97-98 season, the other records were sampled).

**Aggregation via MCMC**

We employed Markov Chain Monte Carlo (MCMC) to estimate the multiplicative factors for each pathogen.^13^ We assume the unobserved true positivity rate $\bar{v}_{i}(t)$ for each virus $i$ ($i=1,\cdots,6$) is Gaussian distributed: $\bar{v}_{i}\left( t \right)\sim\mathcal{N}(v_{i}\left( t \right),\sigma_{v,i}^{2}(t))$ (the noise is additive), where $v_{i}(t)$ is the observed positivity rate at week $t$, and $\sigma_{v,i}^{2}(t)$ is the observational error variance in Eq. 7. (Note that the OEV setting for each pathogen is consistent in model fitting, forecasting and MCMC.) The assumption of a Gaussian distribution for the positivity rate $\bar{v}_{i}\left( t \right)$ is motivated by the fact that a weighted average of Gaussian distributions is still Gaussian. This is appealing as the distribution of ILI can be obtained from the distributions of the individual viral components, which facilitates the computation of likelihood in the MCMC. ILI, as a linear combination of six Gaussian random variables ($ILI(t)=\sum_{i=1}^{6} w_{i}\bar{v}_{i}\left( t \right)$), is also Gaussian distributed: $ILI(t)\sim\mathcal{N}(\sum_{i=1}^{6} w_{i}v_{i}(t),\sum_{i=1}^{6} w_{i}^{2}\sigma_{v,i}^{2}(t))$. Under this assumption, the log likelihood of observing the released ILI data up to the forecast week can be computed using the probability density function of Gaussian distributions. The objective of the MCMC algorithm is to estimate the posterior distribution of each multiplicative factor.

The prior distribution for each multiplicative factor was set as a uniform distribution between 0 and 1. Several other prior ranges were tested; we found that the range [0, 1] is wide enough to cover the estimated posterior distributions of all six multiplicative factors. Starting from a set of multiplicative factors randomly drawn from the prior distributions, a Metropolis algorithm was applied. At each update, a new set of multiplicative factors was obtained by sequentially perturbing $w_{i}$. Specifically, we used a proposal density $w_{i}\left( n+1 \right)\mathcal{\sim N(}w_{i}\left( n \right), \sigma^{2})$, where $w_{i}(n)$ is the multiplicative factor at step $n$ for pathogen $i$, and $\mathcal{N}$ is a Gaussian distribution with a standard deviation $\sigma=0.01$. Whether this update was accepted was determined by the change of log likelihood. In particular, the acceptance ratio is calculated as $\alpha=\min\left( 1,\exp\left( ll_{new}-ll_{old} \right) \right)$, where $ll_{new}$ and $ll_{old}$ are the log likelihoods for the updated and old multiplicative factors. We repeated the MCMC updates $2\times{10}^{4}$ times, dropped the first half as burn-in, and selected samples every 10 steps (1,000 samples) as the final posterior distribution.

We aggregated the forecasts for six pathogens to generate ILI predictions. We randomly matched the 1,000 forecast trajectories for each pathogen with each other, and used samples of the $w_{i}$ posteriors to aggregate the predictions. Note that, the MCMC algorithm only uses released data (i.e., no data from the forecast period is employed). As a result, the estimated multiplicative factors are derived independent of positivity rates during the forecast period.

We observed large variations in the estimated multiplicative factors across seasons (S6 Figure). The variation of multiplicative factors across seasons may be due to a number of issues. First, for pathogens with low positivity rates (e.g., non-circulating influenza strain and PIV12 in several seasons), the multiplicative factors will not be well constrained by the MCMC, as the aggregated time series is not sensitive to those multiplicative factors. This outcome is clearly seen in the elevated and broad distributions for the PIV12 factors in 1998, 2000, 2002 and 2004 (S6 Figure), during which the PIV12 positivity rate was much lower than in other seasons. Second, changes of sampling methods across seasons may also lead to variability. Such changes may affect the magnitude of the positivity rate for a given pathogen over time. For instance, in early seasons the magnitude of PIV12 positivity rate is generally lower than in more recent seasons. The spread of the estimated multiplicative factors also depends on the magnitude of positivity rate signals. For pathogens with low signal, the MCMC can’t constrain the multiplicative factors well (e.g., multiplying a signal of 0.01 by 0.5 or 0.1 does not make much difference in the aggregated ILI). The spread of the distribution thus reflects the uncertainty of the estimation. The magnitude of RSV positivity rate is in general high across seasons, which supports a lower uncertainty. In contrast, signals for some pathogens remain very low in certain seasons. Multiplicative factors for those pathogens thus have a wider spread.

**Post-processing**

The aggregated ILI forecast can be further improved by implementing a post-processing procedure. Similar post-processing approaches have been used in the forecast of ILI [14] and the presidential election [15]. The final ILI forecast is represented as

$$ILI\left( t \right)=\sum w_{i}\bar{v}_{i}\left( t \right)+\varepsilon_{s}\left( t \right)+\varepsilon_{c}\left( t \right). (15)$$

Here the random variable $\varepsilon_{s}\left( t \right)$ is the systematic bias across all seasons, and $\varepsilon_{c}(t)$ is the discrepancy specific to the current forecast. We assume $\varepsilon_{s}\left( t \right)$ follows a Gaussian distribution: $\varepsilon_{s}\left( t \right)\mathcal{\sim N(}\mu\left( t \right), \mu\left( t \right)^{2}/k_{s}^{2}).$ Because the discrepancy is the same across seasons, we set $\mu\left( t \right)$ as the average residual of regression, as shown in S3 Figure, and fixed it during forecast. The standard deviation was allowed to vary, controlled by a parameter $k_{s}$. Note that the residual in S3 Figure captures the decrease of ILI during the Christmas-New Year holiday in most regions.

For the discrepancy specific to the current forecast, $\varepsilon_{c}\left( t \right)$, we also assume it is Gaussian distributed: $\varepsilon_{c}\left( t \right)\mathcal{\sim N(}\delta\left( t \right), \delta\left( t \right)^{2}/k_{c}^{2})$, where $\delta(t)$ is the mean forecast-specific discrepancy at week $t$. In previous studies, $\varepsilon_{c}\left( t \right)$ was modeled as a reverse random walk [14,15]. Here we used another approach: we first modeled the mean value $\delta(t)$, and then generated $\varepsilon_{c}\left( t \right)$. As indicated in the reverse random walk approach, the discrepancy at the last week $t_{L}$ is usually small. As a result, we assume $\delta(t_{L})$ follows a Gaussian distribution around 0: $\delta\left( t_{L} \right)\mathcal{\sim N}\left( 0,\sigma_{\delta\left( t_{L} \right)}^{2} \right)$. We further assume that $\delta(t)$ changes smoothly from the current week $t_{f}$ to the last week $t_{L}$. Instead of an autoregressive reverse random walk [14,15], we chose a flexible functional form for $\delta(t)$ with a smaller number of parameters:

$$\delta\left( t \right)=\delta\left( t_{f} \right)+\left( \delta\left( t_{L} \right)-\delta\left( t_{f} \right) \right)\left( \frac{t-t_{f}}{t_{L}-t_{f}} \right)^{\theta}, (16)$$

where the parameter $\theta$ controls the curvature of the smooth change from the average discrepancy at forecast week $t_{f}$, $\delta\left( t_{f} \right)$, to $\delta\left( t_{L} \right)$ during the last week. We set the discrepancy for ILI at forecast week $t_{f}$ as $\delta_{0}=ILI\left( t_{f} \right)-\sum w_{i}\bar{v}_{i}\left( t_{f} \right)-\mu\left( t_{f} \right)$, which measures the discrepancy from the aggregated ILI to the observed ILI at the forecast week. Because the observed $\delta_{0}$ is subject to observational error, we assume $\delta\left( t_{f} \right)\mathcal{\sim N}\left( \delta_{0},\delta_{0}^{2}/k_{0}^{2} \right)$.

To sum up, the procedure to generate $\varepsilon_{c}\left( t \right)$ proceeds as follows: 1) draw $\delta\left( t_{L} \right)\mathcal{\sim N}\left( 0,\sigma_{\delta\left( t_{L} \right)}^{2} \right)$; 2) draw $\delta\left( t_{f} \right)\mathcal{\sim N}\left( \delta_{0},\delta_{0}^{2}/k_{0}^{2} \right);$ 3) calculate $\delta(t)$ using Eq. 16; 4) draw $\varepsilon_{c}\left( t \right)\mathcal{\sim N(}\delta\left( t \right), \delta\left( t \right)^{2}/k_{c}^{2})$.

Having generated 1,000 samples of $\varepsilon_{s}\left( t \right)$ and $\varepsilon_{c}\left( t \right)$, the forecast ensemble becomes

$$ILI^{j}\left( t \right)=\sum w_{i}^{j}\left( t \right)v_{i}^{j}\left( t \right)+ \varepsilon_{s}^{j}\left( t \right)+\varepsilon_{c}^{j}\left( t \right). (17)$$

Here $ILI^{j}\left( t \right)$ is the $j$th aggregated forecast for ILI; $w_{i}^{j}(t)$ is the $j$th sample of the multiplicative factor for virus $i$; $v_{i}^{j}(t)$ is the $j$th predicted positivity rate for virus $i$; $\varepsilon_{s}^{j}\left( t \right)$ is the $j$th sample of systematic discrepancy; and $\varepsilon_{c}^{j}\left( t \right)$ is the $j$th sample of forecast-specific discrepancy. Usually the forecast ensemble $\{ILI^{j}(t)\}$ is too broad with an excessive variance. So in the final step, we redistributed the forecast ensemble to calibrate its spread. Specifically, the final forecast ensemble is

$$ILI^{j}\left( t \right)=\bar{ILI}\left( t \right)+c_{ILI}\left( ILI^{j}\left( t \right)-\bar{ILI}\left( t \right) \right), (18)$$

where $\bar{ILI}\left( t \right)$ is the mean forecast value and $c_{ILI}$ controls the spread.

In summary, the post-processing involves 6 parameters: $k_{s}$ for the variance of systematic discrepancy, $k_{c}$ for the variance of forecast-specific discrepancy, $k_{0}$ for the variance of mean discrepancy $\delta(t_{f})$ at the forecast week, $\sigma_{\delta\left( T \right)}$ for the variance of mean discrepancy $\delta(t_{L})$ at the last week, $\theta$ for the curvature of smooth change of $\delta\left( t \right)$, and $c_{ILI}$ for the spread of ILI forecast. We optimize these parameters simultaneously using SA to maximize the log score for 1- to 4-week ahead prediction. The ranges for these parameters are: $k_{s}\in\left[ 0,10 \right]$, $k_{c}\in[0,10]$, $k_{0}\in[0,10]$, $\sigma_{\delta\left( t_{L} \right)}\in[0,0.05]$, $\theta\in[0,10]$, $c_{ILI}\in[0.1,2]$.

The effects of adjusting systematic discrepancy, forecast-specific discrepancy and redistribution are presented in Fig. 3A. The post-processing procedure improves the forecast accuracy and precision (Fig. 5). Examples of ILI forecasts at different phases of the 2010-2011 through 2013-2014 outbreak are shown in S11, S12, S13 and S14 Figures.

**Forecasting ILI as a single pathogen**

We performed a baseline method that simulates ILI as a single infectious agent to compare the multi- and single-pathogen forecasts. We used a humidity-forced SIRS model to describe the transmission process, and applied a data assimilation algorithm – the Ensemble Adjustment Kalman Filter (EAKF) [6] – to estimate the model state using released ILI data. With EAKF, the distribution of the model state was represented by an ensemble of state vectors, which were iteratively updated to fit observations. These ensemble members were then integrated forward in time to generate forecasts. In this study, we maintained 1,000 forecast trajectories, as for the multi-pathogen approach. This baseline method, coupling a dynamical model with a data assimilation algorithm, has been successfully applied to forecast a range of infectious diseases, and achieved satisfactory performance in the real-time prediction of ILI in the United States [16-18]. In implementation, the initial ranges of model variables and parameters are set as follows: $N={10}^{5}$, $S\in\left[ 0.3,0.6 \right]N$, $I\in\left[ 0,0.01 \right]N$, $R_{0max}\in\left[ 1.3, 4 \right]$, $R_{0min}\in\left[ 0.8, 1.3 \right]$, $L\in\left[ 180, 730 \right]$ days, $D\in[2,7]$ days. The scaling parameters are set as 1 in all regions. The OEV is set as $OEV_{t}={10}^{-5}+{(\sum_{t^{'}=t-2}^{t} ILI\left( t^{'} \right)/3)}^{2}/5$.

**Evaluation of retrospective forecasts**

The log score and forecast error at different forecast weeks for seven targets are reported in S15 Figure. The multi-pathogen forecast outperforms the single-pathogen forecast in most forecast weeks. Reliability plots for seven targets are reported in Fig. 5. The calibration for near-term targets and peak intensity is substantially improved.

In order to examine whether the multi-pathogen forecasting algorithm outperforms the baseline approach, we performed a Wilcoxon signed-rank test. The Wilcoxon signed-rank test is a non-parametric statistical test that compares two paired samples (here, paired log scores or MAEs generated by both examined methods for the same location at the same forecast week) to assess whether their population mean ranks differ. We performed a two-sided test to return a p-value indicating whether the multi-pathogen forecasting significantly outperforms the baseline. We calculated the p-values for each of seven targets using the *signrank* function in MATLAB.

**Reference**

1. Shaman J, Karspeck A. Forecasting seasonal outbreaks of influenza. *Proc Natl Acad Sci U S A* 2012; **109**: 20425–30.
2. Shaman J, Pitzer VE, Viboud C, Grenfell BT, Lipsitch M. Absolute humidity and the seasonal onset of influenza in the continental United States. *PLoS Biol* 2010; **8**: e1000316.
3. Shaman J, Kohn M. Absolute humidity modulates influenza survival, transmission, and seasonality. *Proc Natl Acad Sci U S A* 2009; **106**: 3243–3248.
4. Cosgrove BA, Lohmann D, Mitchell KE, Houser PR, Wood EF, Schaake JC, Robock A, Marshall C, Sheffield J, Duan Q, Luo L. Real-time and retrospective forcing in the North American Land Data Assimilation System (NLDAS) project. Journal of Geophysical Research: Atmospheres. 2003 Nov 27;108(D22).
5. Ionides EL, Bretó C, King AA. Inference for nonlinear dynamical systems. *Proc Natl Acad Sci U S A* 2006; **103**: 18438–43.
6. Anderson JL. An ensemble adjustment Kalman filter for data assimilation. *Mon Weather Rev* 2001; **129**: 2884–903.
7. Pei S, Morone F, Liljeros F, Makse H, Shaman J. Inference and control of the nosocomial transmission of methicillin-resistant Staphylococcus aureus. *elife* 2018; **7**: e40977.
8. Molteni F, Buizza R, Palmer TN, Petroliagis T. The ECMWF ensemble prediction system: Methodology and validation. *Q J R Meteorol Soc* 1996; **122**: 73–119.
9. Toth Z, Kalnay E. Ensemble forecasting at NCEP and the breeding method. *Mon Weather Rev* 1997; **125**: 3297–319.
10. Pei S, Cane MA, Shaman J. Predictability in process-based ensemble forecast of influenza. *PLoS Comput Biol* 2019; **15**: e1006783.
11. Kirkpatrick S, Gelatt CD, Vecchi MP. Optimization by simulated annealing. *Science* 1983; **220**: 671–680.
12. Viboud C, Boëlle PY, Carrat F, Valleron AJ, Flahault A. Prediction of the spread of influenza epidemics by the method of analogues. *Am J Epidemiol* 2003; **158**: 996–1006.
13. Gelman A, Stern HS, Carlin JB, Dunson DB, Vehtari A, Rubin DB. Bayesian data analysis. Chapman and Hall/CRC (Boca Raton, FL); 2013.
14. Osthus D, Gattiker J, Priedhorsky R, Del Valle SY. Dynamic Bayesian Influenza Forecasting in the United States with Hierarchical Discrepancy. *Bayesian Anal* 2019; **14**: 261–312.
15. Linzer DA. Dynamic Bayesian forecasting of presidential elections in the states. *J Am Stat Assoc* 2013; **108**: 124–34.
16. Biggerstaff M, Alper D, Dredze M, et al. Results from the centers for disease control and prevention’s predict the 2013–2014 Influenza Season Challenge. *BMC Infect Dis* 2016; **16**: 357.
17. Biggerstaff M, Johansson M, Alper D, et al. Results from the second year of a collaborative effort to forecast influenza seasons in the United States. *Epidemics* 2018; **24**: 26–33.
18. McGowan CJ, Biggerstaff M, Johansson M, et al. Collaborative efforts to forecast seasonal influenza in the United States, 2015–2016. *Sci Rep* 2019; **9**: 683.
