# Supplementary material for: Aggregating forecasts of multiple respiratory pathogens supports more accurate forecasting of influenza-like illness
Source: PLoS Comput Biol. 2020 Oct 22;16(10):e1008301. doi: 10.1371/journal.pcbi.1008301 (PMC7608986; doi:10.1371/journal.pcbi.1008301)
Supplement: S1 Table — We measure the residual of model fitting to ILI using the sum of absolute discrepancies at all weeks in each season and location. The first numbers are the residuals of multi-pathogen model fittings, and the latter ones are obtained from single-pathogen model fittings. The smaller fitting residuals are in bold. In general, the multi-pathogen model fittings have smaller residuals in most seasons and locations. (DOCX) [file pcbi.1008301.s002.docx]

**S1 Table. Residuals of multi-pathogen and single-pathogen model fittings to ILI.** We measure the residual of model fitting to ILI using the sum of absolute discrepancies at all weeks in each season and location. The first numbers are the residuals of multi-pathogen model fittings, and the latter ones are obtained from single-pathogen model fittings. The smaller fitting residuals are in bold. In general, the multi-pathogen model fittings have smaller residuals in most seasons and locations.

| Season | National | Region 1 | Region 2 | Region 3 | Region 4 | Region 5 | Region 6 | Region 7 | Region 8 | Region 9 |
| --- | --- | --- | --- | --- | --- | --- | --- | --- | --- | --- |
| 97-98 | **0.16**/0.27 | 0.26/**0.22** | 0.75/**0.68** | **0.27**/0.31 | 0.20/0.20 | **0.15**/0.24 | **0.30**/0.61 | **0.21**/0.25 | **0.35**/0.39 | **0.33**/0.48 |
| 98-99 | **0.19**/0.51 | **0.19**/0.30 | **1.30**/1.38 | **0.40**/0.84 | **0.11**/0.21 | **0.31**/0.44 | **0.71**/0.80 | **0.21**/0.33 | 0.18/0.18 | **0.37**/0.85 |
| 99-00 | **0.14**/0.20 | 0.12/**0.08** | 0.46/**0.38** | 0.52/**0.46** | 0.18/**0.16** | 0.16/**0.12** | 0.43/**0.26** | **0.19**/0.21 | 0.16/**0.12** | **0.31**/0.62 |
| 00-01 | **0.12**/0.21 | **0.11**/0.15 | 0.27/**0.26** | **0.30**/0.39 | **0.17**/0.21 | **0.07**/0.08 | 0.37/**0.28** | **0.20**/0.23 | **0.09**/0.11 | **0.20**/0.51 |
| 01-02 | **0.13**/0.31 | **0.10**/0.11 | 0.19/**0.16** | **0.44**/0.57 | **0.10**/0.11 | **0.21**/0.26 | **0.25**/0.34 | 0.32/**0.27** | **0.11**/0.14 | **0.62**/0.87 |
| 02-03 | **0.11**/0.16 | **0.08**/0.09 | **0.22**/0.32 | **0.11**/0.18 | **0.12**/0.14 | **0.21**/0.29 | **0.23**/0.32 | **0.12**/0.15 | 0.09/0.09 | **0.28**/0.38 |
| 03-04 | 0.28/**0.17** | 0.18/**0.12** | **0.19**/0.39 | **0.18**/0.26 | 0.15/0.15 | 0.29/**0.19** | 0.51/**0.40** | 0.57/**0.28** | 0.25/**0.22** | **0.28**/0.30 |
| 04-05 | **0.12**/0.16 | **0.10**/0.12 | **0.13**/0.40 | **0.20**/0.46 | **0.15**/0.20 | **0.11**/0.18 | **0.27**/0.42 | **0.13**/0.17 | **0.11**/0.12 | **0.18**/0.22 |
| 05-06 | **0.09**/0.19 | **0.06**/0.12 | **0.18**/0.31 | **0.18**/0.41 | **0.08**/0.14 | **0.10**/0.21 | **0.25**/0.49 | 0.10/**0.08** | **0.20**/0.21 | 0.28/0.28 |
| 06-07 | **0.08**/0.19 | **0.07**/0.13 | **0.08**/0.25 | **0.13**/0.25 | **0.13**/0.20 | **0.09**/0.21 | **0.26**/0.59 | **0.08**/0.13 | 0.10/**0.08** | **0.11**/0.16 |
| 07-08 | **0.12**/0.27 | **0.11**/0.15 | **0.09**/0.21 | **0.10**/0.25 | **0.15**/0.31 | **0.08**/0.26 | **0.24**/0.94 | **0.26**/0.29 | **0.15**/0.17 | **0.20**/0.26 |
| 10-11 | **0.07**/0.19 | **0.04**/0.06 | **0.14**/0.39 | **0.08**/0.12 | **0.11**/0.25 | **0.06**/0.11 | **0.17**/0.45 | **0.16**/0.27 | **0.07**/0.08 | **0.12**/0.35 |
| 11-12 | **0.05**/0.10 | **0.03**/0.06 | **0.05**/0.13 | **0.11**/0.24 | **0.07**/0.13 | **0.06**/0.10 | **0.11**/0.20 | **0.13**/0.22 | **0.04**/0.06 | **0.11**/0.15 |
| 12-13 | **0.09**/0.13 | **0.08**/0.15 | **0.17**/0.20 | **0.13**/0.24 | **0.12**/0.21 | **0.07**/0.19 | **0.16**/0.25 | **0.14**/0.33 | **0.08**/0.10 | **0.16**/0.21 |
| 13-14 | **0.08**/0.15 | **0.07**/0.12 | **0.13**/0.28 | **0.08**/0.18 | 0.17/**0.10** | **0.07**/0.17 | **0.13**/0.35 | **0.08**/0.14 | **0.10**/0.11 | 0.11/0.11 |
