# Supplementary material for: Aggregating forecasts of multiple respiratory pathogens supports more accurate forecasting of influenza-like illness
Source: PLoS Comput Biol. 2020 Oct 22;16(10):e1008301. doi: 10.1371/journal.pcbi.1008301 (PMC7608986; doi:10.1371/journal.pcbi.1008301)
Supplement: Attachment — Submitted filename: Response.pdf [file pcbi.1008301.s023.pdf]

Reviewer #1: Review of “Aggregating forecasts of multiple respiratory pathogens supports more accurate forecasting of influenza-like-illness”

The authors present a well-motivated paper. ILI forecasting has garnered significant public health attention in recent years. ILI, though often treated like a singular entity, is really a collection of numerous respiratory illnesses, including the flu, as the authors state. Moving ILI forecasting models in directions that better align with the ILI data-generating process represents an important step for ILI forecasting. Furthermore, the authors present evidence that forecasting can be improved by such a move.

In principle, I’m bullish on this work. That said, the execution left much to be desired. Numerous issues exist and clarifying questions need to be addressed. By far the most significant issue surrounds the prior specification and sampling of the weights, w_i.

All issues and clarification questions are enumerated below.

Response: We appreciate referee’s efforts in reviewing our manuscript and the many suggestions to improve the work. Our responses to questions are listed below.

Major issues:

1. Do the weights w_i described in lines 166-171 as well as the “Aggregation via MCMC” section of the Supplementary Materials (SM) sum to 1? The authors never say that they do, but they do describe ILI as being the aggregation of 6 pathogens, suggesting that the weights should sum to 1. It seems like they should but not clear if they do. Clarification is needed.

Response: We thank referee for this question. The weights for all pathogens do not necessarily sum to 1. According to the corrected Eq. 3 in the SM, the multiplicative factor $w_i(t) = P_t(T|M)\ /P_t(T|M,V_i)$ quantifies the ratio of the probability of being laboratory tested for respiratory viruses among patients seeking medical attention for any reason to the probability of being laboratory tested among patients seeking medical attention who are infected by pathogen V_i. The sum of those ratios is not necessarily equal to 1. To avoid this confusion, throughout the manuscript and SM, we have replaced “weighted average” and “weighting factors” to “linear combination” and “multiplicative factors”. We also clarified this explicitly in lines 202-205 in the revised manuscript.

2. How are the weights actually estimated? On lines 175-176, the authors say, “We then performed a linear regression to ILI using the fitted curves via MCMC in order to derive estimates for w_i (Fig 1B).” The authors, in the second paragraph of section “Aggregation via MCMC” of the SM go on to say, “The prior distribution for each weighting factor was set as a uniform distribution between 0 and 0.3. Starting from a set of weighting factors randomly drawn from the prior distributions, a Metropolis algorithm was applied. At each update, a new set of weighting factors was obtained by sequentially perturbing w_i.” Because a Uniform [0, 0.3] prior was selected for each weight, that means the posterior must necessarily be between 0 and 0.3, inclusively. That check seems to hold for Figure S5, as the posterior distribution for each weight and each season appears to be between 0 and 0.3. That same check, however, does not hold for the weights in Fig 1B. Specifically, some of the posterior draws for the PIV12 weight exceeds 0.3, which is not possible if they were given a prior

between 0 and 0.3. Explanation is needed. Shouldn't Fig 1B be identical to the 2010 panel of Fig S5? Relatedly, what proposal density is used for the Metropolis algorithm?

Response: Fig. 1B was obtained from a previous version using a prior range [0, 0.4]. In the revised manuscript, we have extended the prior for all pathogens to a wider range [0, 1] (see response to the next question below) and updated the results in the manuscript and SM. The selection of the prior is based on the relative scale of ILI and positivity rates for six pathogens. As the positivity rate of PIV12 is much lower than other pathogens (see S1 Figure), the estimated multiplicative factor for PIV12 is higher and tends to reach the upper bound of the prior. Nevertheless, due to the low absolute value of PIV12 positivity rate (especially around the peak of ILI), this change of prior and w_i for PIV12 does not affect the aggregated time series much. In the Metropolis algorithm, we used a proposal density of $w_i(n+1) \sim \mathcal{N}(w_i(n), \sigma^2)$, where $w_i(n)$ is the multiplicative factor at step n for pathogen i, and $\mathcal{N}$ is a Gaussian distribution with a standard deviation $\sigma = 0.01$. We have added the details describing this proposal density in the revised SM.

3. It's hard to understand what value the posteriors of the weights have, given that they were assigned a seemingly arbitrary prior hard capped above at 0.3 and many of the posterior distributions are bumping up against this arbitrary upper bound (e.g., Fig S5 - PIV12 in 2001, 2002, 2010, and 2011). This suggests that the data actually wants the weight for PIV12 to be larger than 0.3 but it can't because of the selected prior. In general, there is nothing wrong when a posterior bumps up against a prior upper or lower bound when that upper or lower bound represents a physical constraint. When a prior bumps up against an arbitrary bound, however, that is a clear sign of prior misspecification. In such a case, a new prior should be selected that pushing those bounds out as the fits and likely the forecasts are suffering from this arbitrary 0.3 upper bound choice. It seems a more physical and elegant solution would be to assign a Dirichlet prior to the weight vector (all 6 weights jointly). A Dirichlet prior (and Metropolis-Hastings proposal) will guarantee that 1) all weights are positive and 2) all weights will sum to 1. Thus, if a weight bumps up against its lower (0) or upper (1) bound, that will be alright because those bounds represent physical lower and upper weight limits. If the authors choose not to change their priors and keep the analysis as is, they will need to provide extremely strong arguments as to why the weights cannot exceed 0.3.

Response: We thank reviewer for the suggestion of using a Dirichlet prior. As we explained in the response to the first question, the multiplicative factors do not necessarily sum to 1. As a result, the six factors do not follow a Dirichlet distribution. The prior issue mostly affects the multiplicative factor for PIV12. This is due to the low PIV12 positivity rates relative to other pathogens (see S1 Figure). For the seasons in which the posterior PIV12 factor bumps up against the prior upper bound, the positivity rate for PIV12 in the first few weeks is extremely low (around 0.01 as shown in S1 Figure). This low positivity rate forces the PIV12 multiplicative factor to a high value in order to fit the ILI time series during the first few weeks, when the activities of other pathogens are still low. In the revision, we tested several wider prior ranges, and found that a range of [0,1] is wide enough to cover the posteriors for all six pathogens (S6 Figure in the revised SM). Results in the manuscript and SM were updated using this new prior. We note that, the aggregated time series are not substantially changed due to the low PIV12 positivity rates (multiplying 0.01 by 0.5 or 0.1 does not make much difference in aggregate).

Issues:

1. Throughout the manuscript, the authors say their models are "accurate" in an absolute sense (e.g., line 178; lines 218-220). However, the authors never define what "accurate" means in an absolute sense leaving the reader to define this for themselves. This is challenging to do as some of the language the author's use deviates from my interpretations. To me, for instance, the red line in the Strain B panel of Fig 1A deviates from the observations roughly as significantly as the yellow line (single agent) does in Fig 1C. However, the authors say, "The mechanistic models accurately reproduced the positivity rates of each pathogen (Fig 1A)" (line 178) to describe the Strain B fit while simultaneously describe the single pathogen fit in Fig 1C as having, "large discrepancies from observations" (line 183). If the authors choose to describe their fits as "accurate" in an absolute sense, they need to provide an unambiguous definition of what "accurate" means and then demonstrate their fits either do or do not meet that unambiguous definition. Otherwise, the authors should refrain from such absolute claims.

Response: We agree with the reviewer that, when comparing the fitting/forecasting for a single time series, "accurate" is not suitable without a clear definition. We therefore removed the term "accurately/accurate" in the revised manuscript whenever there is no objective criterion to define accuracy. The term "accurate" for forecasts in the manuscript is used to compare the performance of multi-pathogen forecasting and the baseline averaged over multiple seasons and regions, which can be quantified by objective evaluation metrics.

2. Table 1: The percentages within a row in Table 1 do not sum to 100%. Are they supposed to? It seems like they should. I don't know how to interpret them if they don't.

Response: We thank reviewer for pointing this out. According to our definition of contribution – $\sum_{t=1}^{52} w_i v_i(t) \,/\, \sum_{t=1}^{52} ILI(t)$, the denominator (i.e., observed ILI) may not equal exactly to the sum of numerators (i.e., the aggregated time series) due to imperfect fitting. In the revised manuscript, we changed the definition of this contribution to $\sum_{t=1}^{52} w_i v_i(t) \,/\, \sum_{i=1}^{6} \sum_{t=1}^{52} w_i v_i(t)$, which guarantees that the percentages within a row sum to 100%. Please find these changes in Table 1 of the revised manuscript.

3. Lines 230 and 240: Please provide more detail regarding how the Wilcoxon signed-rank test was performed. Its appropriateness is unclear based on the information provided.

Response: In the Wilcoxon signed-rank test, we compared two paired samples, i.e., paired log scores or MAEs generated by both the multi-pathogen forecasting and baseline for the same location at the same forecast week, to assess whether their population mean ranks differ. We performed a two-sided test to return a p-value indicating whether the multi-pathogen forecasting significantly outperforms the baseline. We calculated the p-values for each of seven targets using the *signrank* function in MATLAB. In the revised manuscript, we added details of this Wilcoxon signed-rank test in the SM.

4. Line 257: A conclusion of this work is that for the 2010-2011 season, flu strains account for up to 80% of ILI of the six examined viruses. How does this 80% estimate compare to the author's previous work with ILI+, which is a scaling of ILI by the proportion of ILI patients testing positive for influenza. At first blush, 80% seems high.

Response: The 80% contribution from influenza is only for the time point when ILI peaks, see Fig. 6 in the revised manuscript around week 18. "*In this example, influenza strains account for up to 80% of ILI attributed to the six examined viruses* ***during the period of peak ILI***." The overall contribution from influenza for the whole season is much lower. Also, this is only

the relative contribution among the six examined pathogens, which we have stressed explicitly. We modified the statement to "In this example, during the period of peak ILI, influenza strains account for the majority of ILI attributed to the six examined viruses" in the revised manuscript.

5. SM second equation: The fraction should be flipped, with P(TIM) in the denominator. This means the fraction in the third equation also needs to be flipped and the interpretation of the weight is backwards. In general, the "Disaggregation of ILI" section should be closely examined and updated in light of the error in the second equation.

Response: We thank reviewer for pointing out this error. We have corrected this error and updated the "Disaggregation of ILI" section. Note that this error only affects the interpretation of the meaning of multiplicative factors w_i and does not impact the forecasting algorithm. Please see changes in the SM.

6. Table S1: It's not intuitive to me why the multi-pathogen fit should ever be worse than the single-pathogen fit. Isn't the model space of the single-pathogen contained within the more flexible multi-pathogen model space? Said another way, the multi-pathogen fit can take any form the singe-pathogen fit can take, but the single-pathogen fit cannot take any form the multi-pathogen fit can take. If this is true, it's unclear why, when fitting (not forecasting) the multi-pathogen fit should ever be worse than the single-pathogen fit.

Response: Compared with the single-pathogen fitting to ILI, the multi-pathogen fitting has additional constraints – the curve for each pathogen needs to fit observed positivity rates (as shown in Fig. 2A in the revised manuscript). In other words, components in the multi-pathogen fitting are not flexible to take any form. These additional constraints in fact make the multi-pathogen fitting to ILI even more challenging than the single-pathogen fitting, as the shape of each component is already fixed by these observed positivity rates. Additionally, if the positivity rate data are noise in some regions/seasons, it could corrupt the multi-pathogen fitting. Even with those constraints and limitations, in most cases, the multi-pathogen fitting still has a smaller residual. Another reason for occasional worse multi-pathogen fitting could be that we have only considered six pathogens in the model. We have added these explanations in line 223-224 in the revised manuscript.

7. Figure S10: The central thesis of the paper is that forecasting ILI as a weighted sum of its constituent pathogens yields better forecasts than forecasting ILI directly as a single pathogen. This conclusion is supported by Fig S10. The authors also demonstrate that forecasting can be further improved by accounting for systematic bias. The glaring hole in the comparison in Fig S10 is the forecasts of the single-pathogen fit (baseline) that accounts for systematic bias. Without this comparison, it's hard to know which modeling technique provides the largest improvement: the multi-pathogen modeling (as is suggested by the thesis of the paper) or the systematic bias modeling. The single-pathogen with systematic bias would be a very welcomed contribution to the paper.

Response: We performed the additional comparison between the baseline single-pathogen forecasting and the baseline with systematic bias correction. The systematic bias correction slightly improves near-term forecasts over the baseline, but degrades predictions for seasonal targets. In the revised manuscript, we reported this additional analysis in the main text and added a new figure, S18 Figure, in the SM.

Minor issues:

1. ILI is “influenza-like illness” not “influenza-like-illness” (e.g., the title and the abstract)

Response: We have corrected this in the revised manuscript.

2. Line 109: In what sense is the perturbing “optimal”? Please provide a bit more context.

Response: We perturbed the initial condition (S and I) of the model to achieve optimal calibration for near-term forecasts (i.e., 50% of observed outcomes fall within the interquartile prediction interval; 95% of observed outcomes fall within the 95% prediction interval; etc.). Specifically, the initial condition is perturbed to minimize the difference between the spread of near-term model trajectories and observations. This difference is quantified by the deviation from the diagonal y=x line in a reliability plot (see Fig. 5 in the revised manuscript for an example). In the revised manuscript, we briefly introduced this concept in the main text and referred readers to S1 Text for more details.

3. Line 110: please use consistent formatting for references (e.g., [28] instead of superscripting).

Response: We have changed this reference format.

4. Lines 162-163: Why are RSV, PIV12, and PIV3 more regular than flu strains? Why do they peak when they do? Is this known? In general, a bit more context should be added to RSV, PIV12, and PIV3 – both what we know and don’t know about their seasonality.

Response: Several observational studies indicate that both RSV and PIV have relatively regular seasonality in temperate regions. In the US, RSV peaks typically occur in early February, and PIV3 and PIV2 usually peaked in April-June and October-November, respectively. The seasonality of RSV was found to be correlated with climate conditions, such as temperature and humidity. However, the exact drivers of the seasonality of RSV and other respiratory viruses and their comparative significance are still debated. In the revised manuscript, we now provide this context in lines 124-129.

5. Lines 166-168: Is there a season index being suppressed in the ILI(t) equation? For instance, is this ILI(t) for season s?

Response: Yes. We omitted the season index for notational convenience. We explained this simplification in the revised manuscript.

6. Line 172: It’s not clear how the dependent clause, “To eliminate the impact of observational error,” is related to the rest of this sentence. How is the fitting of each respiratory pathogen related to the observation error?

Response: We used a smooth fitting curve rather than noisy raw data to perform the linear regression. To avoid confusion, we have removed this clause in the revised manuscript.

7. ILI data is subject to revisions for many weeks after the initial release of the ILI data. Is

backfill accounted for in this work? If not, why not and how do you imagine backfill would impact this work?

Response: We thank the reviewer for bringing up this practical issue. In this study, we did not consider backfill. However, the impact of backfill should be limited as the aggregation uses all available data points, while backfill mostly affects the most recent 2 or 3 weekly data points. The backfill issue can be partly alleviated using nowcasting techniques, which we have developed in other studies. We have added this discussion of this issue to the revised manuscript in lines 385-389.

8. SM last equation in section “Forecasting Influenza”: If you sample a negative positivity rate, do you set it equal to zero (as you know a positivity rate can’t be negative) or do you leave it negative?

Response: We set the negative positivity rate as zero. This is explained in the revised SM.

9. Fig S7 and S11: The 95% prediction intervals are plotted at the x=1 point, not the x=.95 point. Is what’s plotted a 95% prediction interval at the wrong point, or a 100% prediction interval at the right point?

Response: We apologize for this typo. It should be a 100% prediction interval. This is corrected in the revised SM.

10. Table S1: Should “sum of discrepancies” in the Table caption be “sum of squared discrepancies” or “sum of absolute discrepancies”? All reported figures are non-negative, suggesting the sums are not the raw discrepancies.

Response: It should be “sum of absolute discrepancies”. This is clarified in the revised SM.

Reviewer #2: This manuscript builds on a series of prior work from these authors to improve the accuracy of influenza-like illness modelling in the US. It is motivated by the flusight forecasting challenge. In addition to improved forecasting, these studies are fundamentally moving the needle in our epidemiological understanding of influenza and (in this case) of respiratory pathogens in general.

In this manuscript, the authors draw on type-specific regional data to improve their forecasts of national and regional ILI. They use their existing mechanistic models for influenza subtypes and then the method of analogues for the non-flu pathogens. They find consistently increased accuracy of retrospective forecasts with their multi-pathogen approach.

I enjoyed reading the paper and I think it’s a substantial contribution. Forecasting aside, we have very few disease-dynamic studies of multiple pathogens over multiple years. The lower variance of incidence for the other types versus influenza is in itself an interesting result. The authors may wish to comment on the type of immunity and transmissibility of influenza versus the other pathogens. I wonder if influenza has a lowish R0 and long leaky immunity that leads to the variation versus the others?

Response: We thank the referee for the positive feedback on this study and great efforts in evaluating the manuscript. The different seasonality between influenza and other examined respiratory pathogens may be attributed to many factors. For instance, the dynamics of circulating influenza strains depends on pre-exposure to influenza viruses and antigenic drift

that causes waning immunity. RSV mostly infects infants or young children whose maternal immunity has waned over time. The complex interaction between different influenza strains may also contribute to the variation observed in different seasons. In addition, a recent study reported that the virology and host response to RSV and influenza are distinct. While influenza induces robust strain-specific immunity after infection, strain-specific protection for RSV is incomplete. In the revised manuscript, we have added discussions of these issues in lines 244-252.

I have a number of comments around identifying the marginal contribution of different data and approaches to the increased accuracy of the forecasts. However, although I might have made a few different design choices, the merit of the retrospective forecast design (against an established baseline) is that that mis-specification and over-fitting are far less of a worry than a typical likelihood-driven study.

Did the authors explicitly try the method of analogues for the influenza dynamics? I appreciate that they have a lot of experience with the the process models, but given that the toolkit must be to hand in this project, it would be good to know that the process models do do better than the method of analogues and by how much on the metrics used here. Constant scepticism about the merit of prcioess models will result in really good process models at some point!

Response: We thank reviewer for this suggestion. We implemented an additional version of multi-pathogen forecasting in which the components for influenza were generated using the method of analogues. The results are shown in S19 Figure of the revised SM. We found that using the method of analogues for influenza could also support improved forecasting of ILI over the baseline single-pathogen method. However, due to the large variations of influenza activity in different seasons, the improvement using the method of analogues is less than that using process-based models. We report this additional analysis in lines 312-318 of the revised manuscript.

How much did each extra pathogen improve the accuracy? The authors state that more pathogens might be even better, but I wonder if the improvement in accuracy is coming from just a few of the additional non-flus. It would be interesting (and hopefully only resource intensive in computer time rather than human time) to hold each pathogen out in turn and assess the degradation of the forecast.

Response: Thanks for this constructive suggestion. We performed an analysis in which each pathogen was omitted in the multi-pathogen forecasting and compared the degradation of forecasts (S20 Figure). We found that removing any of the six pathogens would lead to a degradation of log score; omitting A/H3 leads to the largest degradation. This is in agreement with our estimated relative contribution to ILI reported in Table 1: A/H3 is the dominant signal in 9 seasons among the 15 seasons examined. In the revised manuscript, we include this analysis in the main text (lines 319-324).

Many of these data will come from multiplex PCR…, but they are being treated as independent observations that contribute to ILI. Are there additional published data from multiplex studies that directly measure the proportion of ILI attributable to each type? Would sample-linked data be able to confirm any of the underlying model parameters. Are there estimates out there for the proportion of ILI in the US due to these 6?

Response: Reporting on the proportion of ILI attributable to different respiratory pathogens has large variations depending on cohort and study locations. For reference, please see Table 1 in Reis & Shaman (Simulation of four respiratory viruses and inference of epidemiological parameters. Infect Dis Model. 2018; 3: 23–34). So far there is still no conclusive reporting on the proportion of ILI in the US due to these pathogens. We have added discussion of this issue and relevant references to the revised manuscript. See lines 335-337.

The charts, both main text and SI are very clear and allow the reader to understand the work quickly.

Response: Thanks.

These authors are part of the ongoing effort with the flusight initiative. Have they used these methods in the competition yet? Might be worth a comment in the discussion without going into too much detail.

Response: Yes. We submitted the multi-pathogen ILI forecasts during the 2019-2020 flusight initiative. The performance is competitive, second to the best performing system from LANL (though note the forecasting period was cut short due to COVID-19). In practice, the performance of the multi-pathogen forecasting is limited by several factors. First, regional level positivity rate data for several pathogens were not available in real time. Consequently, we had to use historical data, which are less specific. Second, even for the national data, the NREVSS surveillance system practices changed, and the real-time data for some pathogens (e.g., RSV) did not agree well with our training dataset. Third, we did not consider ILI backfill and a nowcasting model may be helpful. We have added this to the revised manuscript.

Some specific comments

Line 110; there has been a recent revision in the flusight scoring algorithm, discussed in an exchange of letters in PNAS. https://doi.org/10.1073/pnas.1912147116 Does this paper use the old or the new algorithm. Either is fine, but please state and cite the exchange.

Response: The analysis was done during March and April in 2019, before the PNAS paper published in September 2019. We used the old scoring algorithm. However, in the flusight challenge this season, we used the new scoring algorithm, and achieved competitive performance.

S1 Figure. Its a little difficult to spot patterns here and these are very innovative data. I wonder if scatter plots of each possible pair of observations (so time removed) might give a sense for the amplitude of different pathogens, their variance and their correlation with other pathogens.

Response: Following this suggestion, we added a scatter plot of each possible pair of observations (S2 Figure). We found that, in general, influenza and RSV are positively correlated with each other, and PIV12 and PIV3 are negatively correlated with influenza and RSV. In addition, PIV12 is negatively correlated with PIV3. We reported this result in the revised manuscript.

S2 Figure. I'd put this in the main text. These are the outcome and "exposures" for this study and the patterns here are pretty clear.

Response: We thank reviewer for this suggestion. We have moved S2 Figure to the main text in the revised manuscript.

144; I am more than happy to work in the log score, but maybe include a bit of narrative for the reader about going from score to skill. … -0.5 is correct approx 61% of the time !!

Response: We have added this translation from log score to forecast skill in the revised manuscript.

172; to eliminate the impact of observational error - I couldn't quite understand this.

Response: We used a smooth fitting of the data rather than noisy raw data to perform the linear regression. To avoid confusion, we have removed this clause in the revised manuscript.

178; see comments above, would it be worth testing analogues for influenza as well as the non-flu viruses

Response: We have tested the method of analogues to forecast influenza in the multi-pathogen forecasting and compared its performance with the original multi-pathogen forecasting in which influenza is predicted using process-based models (S19 Figure). Please see lines 307-313 in the revised manuscript.

194; B looks more like the non-flus in this regard?

Response: The variance of the contribution from influenza B is similar to non-influenza pathogens. However, the seasonality of influenza B is more irregular. We comment on this in lines 239-240 of the revised manuscript.

237; % improvement in log scores is a but difficult to interpret. Maybe state the change in log scores or give % changes in skill rather than score?

Response: We report the change in log scores in the revised manuscript.

253; Are there other sources of evidence about proportions of ILI that arise from different pathogens? See comments above.

Response: We have added several references reporting the proportion of ILI that arise from different pathogens in lines 335-337 of the revised manuscript.

276; If arguing for more pathogens being better, would be good to check the incremental impact of each of the pathogens here. See other comments above.

Response: We performed an analysis in which each pathogen was omitted in the multi-pathogen forecasting, and compared the degradation of forecasts (S20 Figure). We found that removing any one of the six pathogens does lead to a degradation of log score, and omitting A/H3 leads to the largest degradation.

The SI was very clear. I didn't see the SI data (and Imight have had a play with these!) but assume that it will be uploaded on publication.

Response: The data will be uploaded to GitHub on publication.

Reviewer #3: The main claims of the paper are that modeling ILI as a weighted average of its contributing viral signals leads to more accurate and precise ILI forecasts (relative to a single-pathogen model for ILI), and that such disaggregation also has value from a public health perspective. The originality and innovative nature of this paper rely largely on the decomposition of ILI into component pathogenic contributors. However, it is not entirely clear based on the current intro whether/how such decomposition has been done before. Lines 31-32: "most existing process-based forecasting systems treat ILI as a single infectious agent." Most, but not all? Can you cite some forecasting systems that do not treat ILI as a single infectious agent? How do these differ from your method? (Same in line 62, with the language "almost all".) If there are competitors that also decompose ILI into component pathogens, the specific contributions of this paper relative to those competitors needs to be made explicit.

Response: We thank referee for this comment. To our knowledge, all current process-based forecasting systems treat ILI as a single infectious agent. This has been made explicit in the revised manuscript.

As far as the claim of improved accuracy and precision, the former is thoroughly demonstrated relative to the single-pathogen SIR model and the latter is not rigorously shown. We can see visually in Fig 2 that the 95% CI of the baseline model seems wider for that particular forecast example, but a table or figure giving a comparison of the area of the models' 95% predictive CIs is needed to make this a general claim.

Response: Forecast precision is evaluated using the reliability plot. In the revised manuscript, we have added details on this evaluation of precision and moved the reliability plot to the main text. We also compare the average width of 95% predictive CIs in S17 Figure.

Performance is compared to what seems to be to be a 'bad' baseline that simulates ILI as a single pathogen. How does performance at predicting overall ILI compare to other competitors that don't consider separate pathogens (e.g., the purely statistical top-performing FluSight model Dante)? Obviously the authors' method has advantages from an interpretability standpoint, but if it performs more poorly than a purely statistical model this would imply that the process-based model is still not quite capturing reality very well. Such a result would not invalidate the claims of the paper, or render its contribution irrelevant, but would indicate that further understanding of the process driving the ILI curve is needed.

Response: Dante is not a purely statistical model. It couples a process-based SIR model with a statistical procedure to correct forecast discrepancies. In the 2019-2020 flu season, we have submitted the multi-pathogen ILI forecasting to the flusight challenge. The real-time performance is close to Dante based on our own calculation (the final outcome has not yet been released by the CDC). In practice, we noted that the real-time performance of the multi-pathogen forecasting was limited by several factors. First, regional level positivity rate data for several pathogens were not available in real time; consequently, we had to use historical data, which are less specific. Second, even for the national data, NREVSS surveillance system practices changed, and the real-time data for some pathogens (e.g., RSV) did not

agree well with our training dataset. Third, we did not consider ILI backfill and a nowcasting model may be helpful for improving real-time forecast. Future remediation of these issues may thus further improve the real-time forecasts, which, regardless, will need to be evaluated over multiple seasons.

The authors argue that learning about the trajectories of the individual infectious agents is potentially useful in clinical settings for determining the possible cause of ILI among multiple infectious agents. This claim of pathogen-specific forecasts improving on public health response relative to ILI forecasts alone could be strengthened by a citation. Additionally, the relationship between the sampling protocol for ILI and that of virologic surveillance isn't made entirely clear in the paper. How are virologic surveillance data sampled? Does this sampling protocol vary by year, and does it impact predictions? I'd like to know more about potential biases in the virologic data, and what the underlying population is of the positivity rate (Is it ILI patients? Is it people at the hospital? Are sicker patients more likely to get a virologic test? Etc.).

Response: Infectious disease forecasting is increasingly used to support public health decision-making (Lutz et al. BMC Infe Dis 2019). This has been made particularly apparent during the ongoing COVID-19 pandemic. For intervention planning, forecasting an aggregated ILI signal will not provide much help. Even for typical wintertime cold and flu seasons, pathogen-specific forecasts can provide guidance on more specific intervention measures. For instance, in the 2019-2020 flu season, type B influenza appeared earlier than usual and was abnormally high in several southern states (e.g., Louisiana). Anticipating an early surge of influenza B patients could support a better preparedness to this specific pathogen in healthcare systems. In the revised manuscript, we have added a brief discussion in lines 353-360.

The virologic surveillance data were collected by NREVSS. According to the description on the NREVSS website, participating U.S. laboratories voluntarily report to the CDC the total number of weekly tests performed to detect respiratory viruses, and the weekly aggregate positive tests. Tests include virus antigen detections, isolations by culture, and polymerase chain reaction (PCR) results on a weekly basis. Participating laboratories are spread across the US (https://www.cdc.gov/surveillance/nrevss/labs/index.html), thus the samples are fairly geographically representative. However, as testing is ordered independently by healthcare providers, it is unknown whether sampling is biased to certain groups of patients. In the revised manuscript, a brief introduction of the NREVSS data has been added in lines 96-101.

I am concerned about the assumption that ILI is a weighted sum of the 6 included pathogens. I think that slightly more care needs to be paid to this issue in the main text outside of the discussion section. I'd like to know whether it would be possible in this framework to include a catch-all "other" category. Given that the authors make the argument that accounting for the various pathogen sources of ILI activity has advantages from both a predictability and an interpretability perspective, it seems misguided for the model itself to implicitly not acknowledge that ILI has other contributors than the 6 pathogens having historic data.

Response: We thank reviewer for this advice. We acknowledge that ILI has contributors other than the 6 included pathogens, such as coronavirus, human metapneumovirus, respiratory adenovirus and rhinovirus. However, without pathogen-specific data for those viruses, it is difficult to estimate the relative contribution of the catch-all “other” category. In aggregation, the contributions of neglected pathogens are either included in the forecast

discrepancy, or absorbed by viruses with similar seasonality. In the revised manuscript we stress that the relative contribution is defined only for the examined six pathogens (subsection "Aggregating predictions of multiple pathogens").

In addition to discussing how (or why not) to include an 'other' category, I think more justification beyond data availability could ameliorate some of the concerns of including only those 6 pathogens. Specifically, I want to know what proportion of ILI seems to not be covered by those pathogens having data since 1997. That is, for the years that data are available, what are the positivity rates for the example other-pathogens cited on lines 93-94 (coronavirus, human metapneumovirus and respiratory adenovirus)? If the positivity rates are high, I would worry more that the 6 included pathogens aren't actually what comprises much of ILI. If low, I'm less concerned.

Response: This is a good suggestion. We estimated the relative contribution of nine pathogens to ILI for the 2010-2011 through 2013-2014 seasons by including three additional pathogens: human metapneumovirus (HMPV), respiratory adenovirus (rAD) and rhinovirus (Rhino). The three pathogens account for 21%, 37%, 19% and 31% of the ILI signal in those four seasons (S4 Figure), indicating that the other six pathogens comprise the majority of ILI. Again, we have stated explicitly in the subsection "Relative contribution of each pathogen" that the estimated contribution is only for the six pathogens. In future studies, with the availability of more pathogen-specific data, we plan to include more viruses in the aggregation.

In line 167-168, the authors should be clear about whether or not \sum w_i = 1. Does the weighting factor imply anything about whether some of ILI can be driven by an individual having multiple pathogens? Is this assumption valid? You mention this briefly in the discussion, but a quick note here would also be helpful. I'm also interested in the relationship between the weighting factor and the sensitivity/specificity of the test. If you knew about the TPR/FPR/FNR of the test could this model incorporate that information?

Response: The weights for all pathogens do not necessarily sum to 1. According to Eq. 3 in the SM, the multiplicative factor $w_i(t) = P_t(T|M)\ /P_t(T|M, V_i)$ quantifies the probability of getting a laboratory test among patients seeking medical attention for any reason to the probability of getting a laboratory test among patients seeking medical attention and infected by pathogen V_i. The sum of these ratios is not necessarily equal to 1. Co-infection of respiratory viruses is rare based on our recent study that actively tested 2,685 participants recruited at a New York City tourist attraction (Birger et al. mSphere (2018): e00249-18). Information on test sensitivity/specificity is not available for this study. If available, we can use it to inform the observation error variance (OEV) of the positivity rate data. For instance, test results with high sensitivity and specificity will be assigned a low OEV, which gives more credibility to those observation and would impact the final estimated multiplicative factor w_i. Discussions of these issues have been added to the revised manuscript.

Figure S5 shows the estimated distributions of weighting factors in different seasons at the national level. It would be useful to also include a similar figure (or a few figures) with season fixed and weighting by region. I'm curious if there is significant regional variability.

Response: New figures (S7 and S8 Figures) have been added in the SM that compare the multiplicative factors for the six pathogens in different regions within the same season. (Note we changed the prior range of w_i in the MCMC from [0. 0.3] to [0, 1], which mostly affects the inferred w_i for PIV12 that has a very low positivity rate.) Within a season, the distributions of

the multiplicative factors in different regions also have variations; however, the relative magnitudes of the influenza types/subtypes remain similar across regions. This analysis is reported in the revised manuscript.

Also in looking at Figure S5 I had the question as to why weighting factors should vary so considerably by season. The authors mention that this is due to contributions themselves being variable. However, it would make more sense to me for the weights to be consistent across seasons, but the positivity rate itself to drive differing pathogenic contributions. The variability of the weighting factors seems to me to perhaps be an artifact of sampling differences across seasons in the virologic data. I think this needs further discussion/consideration.

Response: A number of issues may underlie the variation of the multiplicative factors across seasons. First, for pathogens with low positivity rates (close to zero) (e.g., non-circulating influenza strains and PIV12 in several seasons), the multiplicative factors will not be well constrained by the MCMC, as the aggregated time series is not sensitive to those multiplicative factors. This outcome is clearly seen in the elevated and broad distributions for the PIV12 factors in 1998, 2000, 2002 and 2004 (S6 Figure), during which the PIV12 positivity rate was much lower than in other seasons. Second, changes of sampling methods across seasons may also lead to variability. Such changes may affect the magnitude of the positivity rate for a given pathogen over time. For instance, in early seasons the magnitude of PIV12 positivity rates is generally lower than in more recent seasons. We have added discussions of these issues to the revised SM.

I would also like some comment/discussion from the authors on why the weights for some pathogens in Figure S5 have such small spread (e.g., RSV) while others are hugely variable (e.g., many have box-whisker plots spanning from near-0 to above 0.2). Is there a set of good solutions to the data that can come from essentially 0-weighting a pathogen, while putting all the weight on a different one? Is the set of good solutions highly modal, or is there a smooth trajectory of weights that yield a good fit?

Response: The spread of the estimated multiplicative factors depends on the magnitude of the positivity rate signal. As discussed above, for pathogens with low signal (e.g., non-circulating influenza and PIV12 in some early seasons), the MCMC can’t constrain the multiplicative factors well. (Multiplying a signal of 0.01 by 0.5 or 0.1 does not make much difference in the aggregated ILI.) The spread of the distribution thus reflects the uncertainty of the estimation. The magnitude of RSV positivity rate is in general high across seasons, which supports a lower uncertainty. In contrast, signals for some pathogens remain very low in certain seasons. Multiplicative factors for those pathogens thus have a wider spread.

To evaluate the impact of removing pathogens, we performed an analysis in which each pathogen was omitted in the multi-pathogen forecasting, and compared the degradation of the ILI forecasts (S20 Figure). We found that removing any one of the six pathogens would lead to a degradation of log score, and that omitting A/H3 leads to the largest degradation. Basically, including more pathogen improves the forecasts. We have included this new analysis in the revised manuscript.

A handful of smaller comments:
- Figure 4 is beautiful!!

Response: Thank you.

- Is Figure S6 a remarkable example (particularly good or bad)? Having just one such example makes me wonder why it was chosen, so perhaps include a few more and note the performance relative to overall average performance.

Response: More forecast examples have been added to the SM. Specifically, forecasts in seasons 2010-2011 through 2013-2014 at different weeks are shown. Based on these examples, forecast performance looks stable over different seasons.

- In line 109-110 what is the superscript 28 referring to?

Response: It is a reference. We have changed this to the correct format in the revised manuscript.

- I appreciate the authors noting that the particular structure of the model (modeling ILI as an aggregation of component parts) isn't unique to pathogens but could be used to aggregate forecasts by age group or geography.

Response: Thanks.

- Is the code available? For reproducibility it would be helpful to make this public.

Response: Code will be uploaded to GitHub before publication.
