# Supplementary material for: Aggregating forecasts of multiple respiratory pathogens supports more accurate forecasting of influenza-like illness
Source: PLoS Comput Biol. 2020 Oct 22;16(10):e1008301. doi: 10.1371/journal.pcbi.1008301 (PMC7608986; doi:10.1371/journal.pcbi.1008301)
Supplement: Attachment — Submitted filename: Response.docx [file pcbi.1008301.s024.docx]

Reviewer #3: I thank the authors for their thorough and thoughtful responses to the reviewers! In light of the clarifications/additions, I feel that this manuscript is ready for publication.

I had one small note-- In one of the responses the authors note: “Dante is not a purely statistical model. It couples a process-based SIR model with a statistical procedure to correct forecast discrepancies.” I believe that they are referring to DBM (<https://arxiv.org/abs/1708.09481>, also by Osthus). Unlike DBM, Dante (<https://arxiv.org/abs/1909.13766>) does not rely on a process-based SIR model and is in fact a purely statistical model. In spite of this issue, the authors' response still addressed the underlying question, so thank you for noting the relative performance of your model to Dante.

Response: We apologize for the confusion. Yes, the method we referred to in the response letter is DBM. We will revise our response accordingly should the peer-review file be published along with the paper.

Reviewer #4: The authors have submitted a revised manuscript describing their work on aggregating multiple pathogens to improve ILI forecasting. The initial reviews were generally positive, mostly focusing on clarification and contextualization of the authors' work. The authors have addressed the first round of reviews, and the clarity of the paper is noticeably improved. I do not have any major comments or criticism of the work as is. I think it should be published because it demonstrates a novel ILI forecasting strategy that has the potential for future improvements. I think that the paper's results may be challenging to reproduce verbatim from the supplementary material. Still, because the Authors have said they will post their code to GitHub, I am not concerned. I offer a handful of minor comments.

Response: We appreciate the reviewer’s efforts in evaluating our manuscript. In the revised manuscript, we have addressed all the comments by the reviewer. For reproducibility, the code will be posted to GitHub upon the acceptance of the manuscript.

Comments:

line 68-69: Could you insert a quick definition of "positivity rate" here. Just for completeness.

Response: We have added a clear definition of positivity rate, i.e., the fraction of samples testing positive for a specific respiratory pathogen, in line 69 of the revised manuscript.

line 122: Could "relatively regular outbreaks" be changed to something like "consistent seasonality"? The term regular outbreaks I don't think describes the temporal pattern you are focusing on. Regular outbreaks could mean than they happen annually, whereas you are specifically keying on the regularity of the outbreak time within any given year.

Response: We agree and have replaced “relatively regular outbreaks” with “consistent seasonality” in the revised manuscript.

line 133: Sampled with replacement?

Response: Yes. In the revised manuscript, we have added “with replacement” in the description.

line 140-141: Explicitly state that the prior is uniform(0,1) instead of just describing a range

Response: We have revised the manuscript to “a uniform prior distribution $U(0,1)$” as suggested.

line 145: The word "inferred" is redundant because you infer the true posterior by performing the MCMC sampling

Response: The word “inferred” is removed.

line 355: In this sentence, you say that "forecasting an aggregated ILI signal is insufficient," but forecasting ILI using an aggregation model is what this paper is about. I think you can drop the "aggregated" to emphasize the point that you are trying to make in this paragraph, that is, modeling separate pathogens provides more information and solutions to decision-makers.

Response: Thank you for this suggestion. We have dropped the term “aggregated” in this sentence.

line 636: The units of the "viral signals" in Figure 1 is not explicit. I am assuming it is the observed positivity rate.

Response: Yes. We have added a statement that “the viral signal shown here is the positivity rate of each pathogen” to the caption of Figure 1.

SI Eq. 5: The sign of the I/D term is wrong. It should be negative.

Response: Thank you for pointing out this error. We have fixed this issue in the revised SI.

SI pg. 2: The second paragraph is unclear. What does "free simulation" mean in this context?

Response: This is a good point. We have added an explanation in this paragraph. “Here free simulations were generated using initial conditions and model parameters randomly drawn from the prior ranges, representing typical outbreaks generated from the model. Those synthetic outbreaks were used to compute the typical annual incidence rate of model simulations.” Typical annual incidence rate was then compared with the observed annual incidence rate to estimate the scaling parameter.

SI pg. 4: The assumption of the distribution for $\bar{v}_i(t)$ seems to be implying a Bayesian posterior using an improper prior. Is there a reason for not being explicit about this choice of inference?

Response: The assumption of a Gaussian distribution for the positivity rate $\bar{v}_i(t)$ is motivated by the fact that a weighted average of Gaussian distributions is still Gaussian. This is appealing as the distribution of ILI can be obtained from the distributions of the individual viral components, which facilitates the computation of likelihood in the MCMC. In the revised SI, we have added this discussion on the choice of the distribution of $\bar{v}_i(t)$.

SI pg. 4: In paragraph 3, why was the ensemble of forecast trajectories for each pathogen not incorporated into the likelihood when performing the MCMC to sample the multiplicative factors? It seems like you accept/reject the multiplicative factors with one set of $v_i(t)$ and then switching to another set of $v_i(t)$ when performing the forecasting.

Response: When we performed the MCMC to estimate $v_i(t)$, we used the **observed** positivity rate data for the six pathogens, as those observations are the direct measurement of the incidence of each virus. If the ensemble trajectories fit the observed positivity rates well, the $v_i(t)$ estimated from direct observations should generate reliable predictions using the ensemble of forecast trajectories. In other words, it is possible to use the ensemble trajectories in the MCMC to compute the likelihood. But under the assumption that the ensemble trajectories are close to observations, forecasts generated using both methods should be similar. This approach is a choice for model configuration, and the comparison between the two methods could be addressed in a future study.
